# Evaluation Scale or Output Format: The Attentional Mechanism Underpinning Time Preference Reversal

**DOI:** 10.3389/fpsyg.2022.865598

**Published:** 2022-04-14

**Authors:** Yan-Bang Zhou, Qiang Li, Qiu-Yue Li, Hong-Zhi Liu

**Affiliations:** ^1^Department of Social Psychology, Zhou Enlai School of Government, Nankai University, Tianjin, China; ^2^Laboratory of Behavioral Economics and Policy Simulation, Nankai University, Tianjin, China

**Keywords:** time preference reversal, visual attention, evaluation scale, output format, eye-tracking

## Abstract

Time preference reversals refers to systematic inconsistencies between preferences and valuations in intertemporal choice. When faced with a pair of intertemporal options, people preferred the smaller-sooner option but assign a higher price to the larger-later one. Different hypotheses postulate that the differences in evaluation scale or output format between the choice and the bid tasks cause the preference reversal. However, these hypotheses have not been distinguished. In the present study, we conducted a hybrid task, which shares the same evaluation scale with the bid task and shares the same output format with the choice task. By comparing these three tasks, we can figure out the key reason for time preference reversal. The eye-tracking measures reflecting attention allocation, cognitive effort and information search pattern were examined. Results showed that participants' time preference and eye-tracking measures in the hybrid task were similar to those in the choice task, but different from those in the bid task. Our findings suggest that the output format is the core reason for time preference reversal and may deepen our understanding of the mechanisms that underlie time preference reversal.

## 1. Introduction

One of the biggest challenges of decision theories is the inconsistency of decision-making (Roelofsma and Read, 2000; Kim et al., 2012). A typical example is the preference reversal phenomenon, which involves systematic inconsistencies between preferences and valuations (Seidl, 2002). Lichtenstein and Slovic (1971) first demonstrated this phenomenon in decision-making under risk. For instance, one option is “28/36 chance to win $10” (with a high probability of winning a smaller amount of money, P-option), and the other is “3/36 chance to win $100” (with a low probability of winning a larger amount, $-option). When faced with this pair of options, most people choose the higher probability P-option. However, when providing prices for the same options, most people assign a higher price to the larger amount $-option. If the P-option were more valuable than the $-option, the P-option would be sold higher than the $-option based on predicted normative decision theories (Lichtenstein and Slovic, 1973). One option cannot be better or worse than another option simultaneously. This preference reversal phenomenon constitutes a violation of procedure invariance (Tversky et al., 1990; Stalmeier et al., 1997) because the preferences should not change based on their measurements.

Researchers also demonstrated preference reversals in intertemporal choice, called time preference reversal. Intertemporal choice refers to decisions involving tradeoffs among outcomes at different time points (Prelec and Loewenstein, 1991; Frederick et al., 2002). When faced with a pair of intertemporal options, people preferred the smaller-sooner (SS) option but assigned a higher price to the larger-later (LL) one. This kind of time preference reversal pattern is replicated in several studies (Tversky et al., 1990; Bohm, 1994; Stalmeier et al., 1997; Zhou et al., 2021), indicating that the preference gap between elicitations of choices and prices is universal in different decision domains.

Several explanations have been proposed to account for preference reversals, of which the *scale compatibility hypothesis* may be the most prominent (Tversky et al., 1988, 1990; Loomes, 1990; Rubaltelli et al., 2012; Alos-Ferrer et al., 2021). This hypothesis postulates that the attributes compatible with the response scale will be assigned predominant weight because these attributes naturally map onto the response scale (Tversky et al., 1988, 1990; Mellers et al., 1992). Taking the time preference reversal as an example, the bid task wherein the participants provide a price for an intertemporal option is compatible with the outcome information, which is also expressed in monetary values. In this situation, the participants' attention is primarily directed toward the outcome attribute. And they pays less attention to the delay attribute, which therefore receives a lower decision weight. By contrast, the choice task wherein the participants are providing preferred options is not easily mapped onto the response (Rubaltelli et al., 2012). In this situation, the outcome attribute loses some of its salience. Therefore, compared with the choice task, participants in the bid task show more patience (i.e., give more decision weight on outcome attribute). According to the scale compatibility hypothesis, the key reason for preference reversal is that the evaluation scale is different between the choice and the bid tasks. The evaluation scale in the choice task is based on personal preference, whereas the evaluation scale in the bid task is performed based on a monetary scale (Alos-Ferrer et al., 2021).

Researchers also proposed the *strategy compatibility hypothesis* to account for the preference reversals from the dual-system theory perspective (Stalmeier et al., 1997). According to this hypothesis, different output formats can activate different strategies. In the choice task, the dichotomous option is indicated using an ordering or sequence strategy, an intuitive strategy based on the heuristic system (Sloman, 1996; Kahneman and Frederick, 2002; Evans, 2003). Participants' mental procedure is analogous to weighing two objects by a balance (Slovic, 1995). By contrast, in the bid task, a quantitative price is provided by using a more complex mental process, which is a deliberate strategy based on the analytic system. Participants' mental procedure is analogous to measuring an independent object with a sliding scale (Tversky et al., 1990; Slovic, 1995). According to this hypothesis, the key reason for preference reversal is that the output format is different between the two tasks. Quantitative output in the bid task yields greater weight on outcome attribute and a higher level of cognitive effort. In contrast, dichotomous output in the choice task yields a more balanced decision weight and a lower level of cognitive effort.

Researchers have examined the underlying process in preference reversal by using the eye-tracking technique. For instance, Kim et al. (2012) examined the eye movements in the choice and bid tasks in decision-making under risk and found that the preference reversals occurred with a shift in the fixations of the two attributes. Participants in the bid task fixated on the outcome attribute more time than in the choice task, indicating that the decision weight shifted among the two tasks. Zhou et al. (2021) investigated the eye movements in time preference reversals and found that the mean fixation duration and the proportion of gaze time on the outcome attribute varied across the choice and the bid tasks, and the effect of task (choice task vs. bid task) on choices/bids can be mediated by the eye-tracking measures. Their findings suggested a disparity between the decision weight and cognitive effort level in the choice and the bid tasks. In summary, these findings are consistent with the prediction of scale compatibility hypothesis and strategy compatibility hypothesis.

Although the above evidence can help understand the mechanism of preference reversals, the different hypotheses cannot be distinguished by comparing the choice and the bid tasks. The choice and the bid tasks differ in evaluation scale and output format (see Table 1). To examine the effect of the evaluation scale on information processing, Rubaltelli et al. (2012) designed a rating task in which the participants indicated the attractiveness of the options using an 11-point scale. The rating and bid tasks have similar quantitative output but different evaluation scales (see Table 1). The results revealed that pupil dilations, fixation duration, and the number of fixations increased when participants evaluated the options in the bid task rather than the rating task. Their findings suggested that participants were more likely to engage in a deliberative strategy when completing a bid task than a rating task, partially supporting the scale compatibility hypothesis. However, a difference is observed in the quantitative outputs between the two tasks, introducing potential confounding variables. Furthermore, the choice task was not included in their experiment, thereby failing to provide more direct evidence for examining the core reason for preference reversals. Therefore, carefully conducting appropriate experimental tasks appears important for further investigating the mechanisms underlying preference reversals.

**Table 1:** Summary of different tasks in evaluation scale and output format.

**Task**	**Evaluation scale**	**Output format**
Choice task	Personal preference	Dichotomous
Bid task	Monetary scale	Quantitative
Rating task	Attractiveness	Quantitative
Hybrid task	Monetary scale	Dichotomous

Unlike previous research, the present study attempted to conduct a new task by changing the output format in the bid task to test the hypotheses of preference reversals. The new task conducted in the present study combined the monetary evaluation scale of the bid task and the dichotomous output format of the choice task; thus, we called it the hybrid task. In the hybrid task, participants were asked to infer the option with a higher price. In this task, the evaluation scale is based on the monetary scale, but the output format is a dichotomous option. We can distinguish different hypotheses by comparing the hybrid task with the choice and the bid tasks. On the one hand, the choice and hybrid tasks share the same dichotomous output formats but differ in evaluation scale (see Table 1). If the evaluation scale is the key reason for the preference reversal, differences will be observed in behavioral and eye-tracking measures between the two tasks. On the other hand, the bid task and the hybrid task share the same evaluation scale but differ in the output format (see Table 1). If the output format is the key reason for the preference reversal, differences will be observed in behavioral and eye-tracking measures between the two tasks. Following this hypothetical logic, we conducted three tasks to examine the core reason for preference reversal. We used the eye-tracking technique to monitor participants' eye movements during the three tasks. In the present study, we examined the preference reversals in intertemporal choice rather than risky choice, considering that previous research mostly focused on the preference reversals in risky choice (Kim et al., 2012; Rubaltelli et al., 2012; Alos-Ferrer et al., 2016). Few studies have examined preference reversals in intertemporal choice (Zhou et al., 2021).

In addition to the behavioral measures (i.e., choice/bid, response time), we computed three eye-tracking measures to examine the information processing underlying preference reversals. The first measure is the outcome-gaze-proportion (OGP), which is an index of the proportion of time attention that is allocated to the outcome attribute of an intertemporal option (Franco-Watkins et al., 2016; Ashby et al., 2018; Amasino et al., 2019). The values of OGP can reflect the decision weight during choice task (Brandstätter and Körner, 2014) or bid task (Ashby et al., 2012, 2015). The second measure is mean fixation duration (MFD), which refers to the average duration of single fixations in a decision and can reflect the cognitive effort level (Velichkovsky, 1999; Horstmann et al., 2009; Amblee et al., 2017). The deliberate decision strategy usually accompanies long fixations, whereas the intuitive strategy accompanies shorter fixations (Horstmann et al., 2009; Su et al., 2013). The third measure is the search measure index (SMI), which can reflect the degree to which the direction of a search is alternative-based or attribute-based (Bockenholt and Hynan, 1994; Pachur et al., 2013; Liu et al., 2021a). In the intertemporal choice, the deliberative strategy, which is assumed by delay discounting models (Ainslie, 1975; Frederick et al., 2002), posit an alternative-based information search pattern (i.e., higher value of SMI). The heuristic strategy in intertemporal choice (Leland, 2002; Scholten and Read, 2010) usually posits an attribute-based information search pattern (i.e., lower value of SMI).

## 2. Methods

### 2.1. Participants

A sample size of 43 participants has been estimated to provide 95% power to detect a medium effect (Cohen's *f* = 0.25), as assessed using G*Power (Faul et al., 2007). Thus, 53 college students (*M*_*age*_ = 20.9 ± 2.1 years, 21 females) were recruited from a university's human subject pool to participate in the experiment. All participants had corrected-to-normal vision and gave their informed consent before the experiment. The participants received 20 yuan (RMB; approximately US$3.2) in cash for participating and an additional amount (1–10 yuan; approximately US$0.2–$1.6) based on their performance during the experiment. The University'S Ethics Committee approved the study.

### 2.2. Apparatus

We used an Eyelink 1000 plus eye tracker (SR Research, Ontario, Canada) with a head holder (“desktop mode” configuration) and a 17-inch Dell LCD monitor with a refresh rate of 60 Hz to record the eye movements of the participants. The screen has a resolution of 1028 × 764 pixels, with a visual angle of 36° horizontally and 29° vertically. Participants were seated at approximately 58 cm from the screen. Their head movements were minimized with a chin and headrest. Although viewing was binocular, eye movements were recorded from the right eye. The experiment was controlled with SR Research Experiment Builder software. Participants responded to the stimuli using a keyboard.

### 2.3. Stimuli

The stimuli comprised 50 pairs of randomly generated intertemporal options. Each option represented a certain amount of monetary gain after a certain period. All the options involved gains only, and no dominating options existed. The outcomes ranged between 120–990 yuan, and the delays ranged between 11–99 days, see Table S1 in Supplementary Materials (https://osf.io/8w6eq/). The (horizontal/vertical) center-to-center distance between any two pieces of information is greater than 5°, ensuring proper fixation of the information and that peripheral identification of adjacent information is not possible (Rayner, 1998, 2009).

### 2.4. Experimental Task

The present study conducted three tasks: a choice task, a bid task, and a hybrid task. The three tasks presented the same 50 pairs of options on the screen. In the choice task, the participants were asked to choose their preferred option between a pair of SS and LL options. In the bid task, the participants were requested to indicate their exact valuation of these intertemporal options. That is, they would bid an amount that would make them indifferent between getting the intertemporal option or getting the amount they bid. In the hybrid task, participants were asked to indicate the option that they would bid a greater amount on.

**Figure 1:**
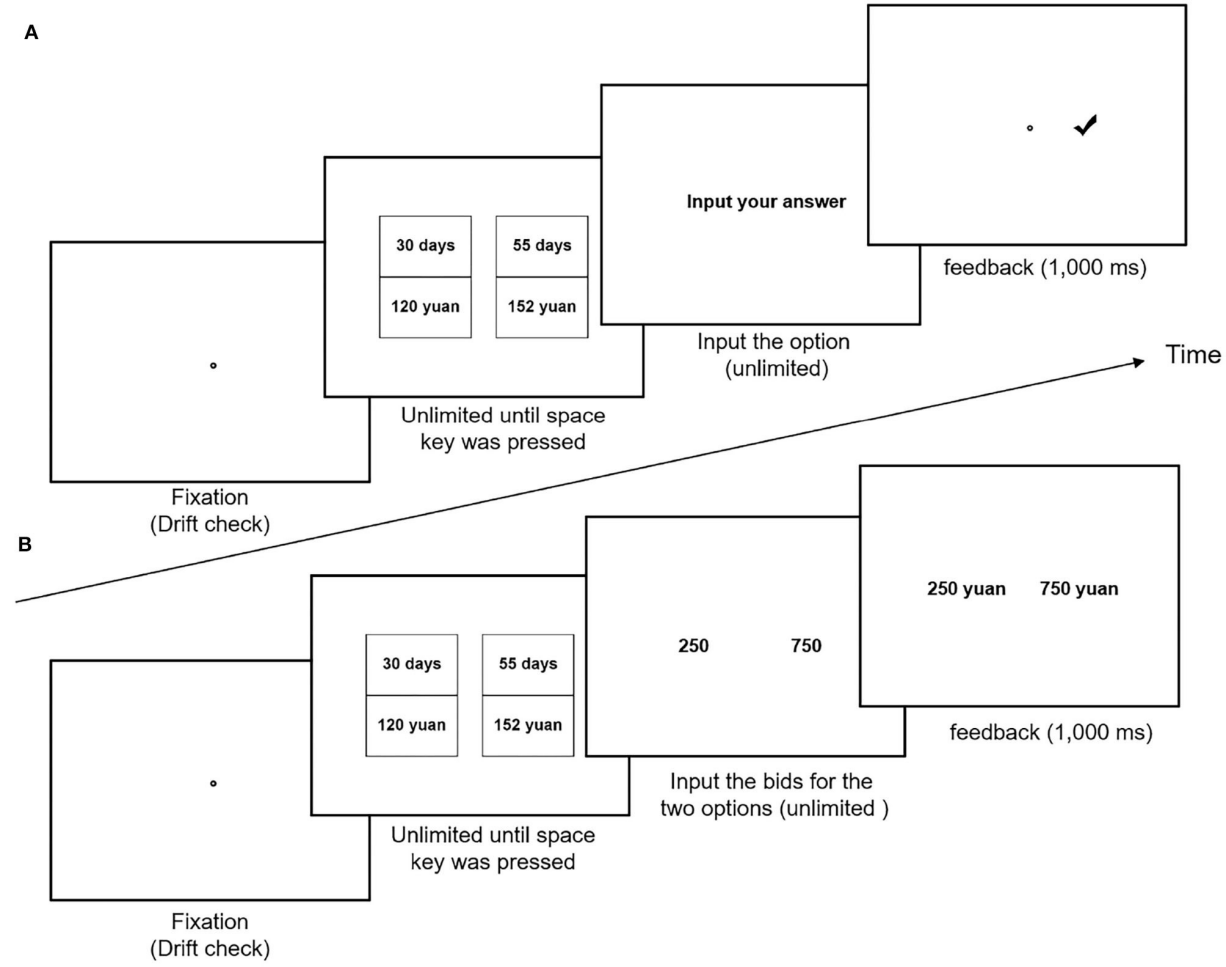
Trial procedure and timing in **(A)** the choice task, the hybrid task, and **(B)** the bid task.

Each participant performed all three of the tasks, but they performed only one task on a given day, with an interval of no less than 3 days between any two subsequent tasks. The order of the tasks was counterbalanced across the participants. Visually identical experimental materials were used in all three tasks (see Figure 1). The placement of attributes (i.e., delay or outcome) was also counterbalanced across participants. Half the participants saw the delay as the top number, and the other half saw the outcome as the top number. The options were presented in randomized order for each participant in each task.

Participants were told that their extra payment would be determined by their performance during the experiment to incentivize further cooperation. They were told that, at the end of the experiment, one of their trials would be randomly selected to receive a real reward at a discount rate. If a trial in the choice task was selected, participants were paid according to their decisions on that trial and received a real delayed reward. If a trial in the bid task was selected, participants were paid using the Becker-DeGroot-Marschak (BDM) method (Becker et al., 1964), which is widely used as an effective incentive in bid tasks (Loomes, 1990; Kim et al., 2012; Ashby et al., 2018; Zhou et al., 2021). If a trial in the hybrid task was selected, participants were then asked to indicate the valuation of the two options just as what they did in the bid task. If they did choose the option that they bid a greater amount, they would be paid the option using the BDM method; otherwise, they would get nothing. This setup can guarantee that they would indeed choose the option that they deemed more valuable in the hybrid task. When paying participants, the delay of the option was multiplied by a rate of 0.1, and the outcome of the option was multiplied by 0.01.

### 2.5. Procedure

After giving their consent, the participants were informed about the experiment and given a brief description of the apparatus. Each participant was calibrated to the eye tracker using a five-dot calibration method at the beginning of the experiment and recalibrated as needed (e.g., if the drift check failed). The maximum error of validation was 0.5 degrees in the visual angle. After the initial calibration, two practice trials were conducted to allow the participants to familiarize themselves with the task.

At the beginning of each trial, a fixation disc was presented at the center of the display. The disc also served as a drift check for the eye tracker. When fixation on that disc was registered, the participants pressed the spacebar to start the presentation of the pair of options. After viewing the options, the participants pressed the spacebar to trigger the reaction screen. In the choice and the hybrid tasks, participants had unlimited time to choose between the two options, pressing “F” to choose the option on the left or “J” to select the option on the right. In the bid task, participants had unlimited time to input their bids for the two intertemporal options using a keyboard and submit their response by pressing the “Enter” key. After participants submitted their responses, feedback was presented for 1,000 ms. See Figure 1 for details.

### 2.6. Data Analysis

#### 2.6.1. Preprocessing of Eye-Tracking Data

The collected eye movement data were analyzed using EyeLink Data Viewer (SR Research, Ontario, Canada). In the three tasks, four non-overlapping, identically sized (10.8° × 7.7° visual angle) rectangular regions of interest around each piece of information were defined. Fixations were defined as periods of a relatively stable gaze between two saccades, but fixations shorter than 50 ms were excluded from the analyses.

#### 2.6.2. Eye-Tracking Measures

As we mentioned above, we used the following three eye-tracking measures to examine the information processing underlying preference reversal. The first measure is the outcome-gaze-proportion (OGP), which is an index of the proportion of gaze time on the outcome attribute of an intertemporal option (Franco-Watkins et al., 2016; Ashby et al., 2018; Amasino et al., 2019). The OGP was computed by


(1) $$\begin{array}{l} OGP=\\ \frac{gaze\;time\;on\;outcome\;attribute}{gaze\;time\;on\;outcome\;attribute+gaze\;time\;on\;delay\;attribute} \end{array}$$

The higher value of OGP indicates that outcome attribute received more attention and greater decision weight than delay attribute.

The second measure is the mean fixation duration (MFD), calculated by adding the duration of all fixations during a trial and dividing the total by the number of fixations. MFD is sensitive to cognitive effort level (Amblee et al., 2017).

The third measure is the search measure index (SMI), which quantifies the degree to which the direction of a search is alternative- or attribute-based (Bockenholt and Hynan, 1994; Pachur et al., 2013; Su et al., 2013; Liu et al., 2021b). The predominance of alternative-based transitions increases with an increasing SMI value (Su et al., 2013). The SMI was computed by


(2) $$\begin{array}{r} SMI=\frac{\sqrt{N}\left[\frac{AD}{N}\left(r_{a}-r_{d}\right)-\left(D-A\right)\right]}{\sqrt{A^{2}\left(D-1\right)+D^{2}\left(A-1\right)}} \end{array}$$

where *A* and *D* denote the number of options and the number of attributes, respectively (i.e., in this experiment, *A* = 2, *D* = 2), *r*_*a*_ and *r*_*d*_ denote the number of alternative-based transitions and attribute-based transitions, respectively, and *N* denotes the number of total transitions. A negative value of SMI indicates a predominantly attribute-based search, and a positive value indicates a predominantly alternative-based search (Pachur et al., 2013).

## 3. Results

Overall, 24 trials (11 trials in the choice task and 13 trials in the bid task) were excluded from analyses because of eye-tracking failures. Thus, 7,926 trials, including 2,639 trials in the choice task, 2,637 trials in the bid task, and 2650 trials in the hybrid task, were included in the analyses.

Data from the experiment reported in this article is publicly available *via* the Open Science Framework (https://osf.io/8w6eq/). We used standard Null Hypothesis Significance Testing and Bayesian statistical methods to analyze our data, using the jamovi software (Şahin and Aybek, 2019). We focus on Bayes Factors (*BF*s) that measure the likelihood of observed data given a particular model. We use the default Cauchy prior with width 0.707 implemented by jamovi in all analyses.

### 3.1. Behavioral Results

#### 3.1.1. Choice/Bid

For each pair of options, we categorized responses in the choice and the hybrid tasks according to whether the participants indicated the SS option (“SS”) or the LL option (“LL”) and categorized responses in the bid task according to whether the participants bid higher on the SS option (“SS”) or bid higher on the LL option (“LL”). No participants bid an equal amount for both options in the bid task. We calculated the proportions of each category for each participant.

**Figure 2:**
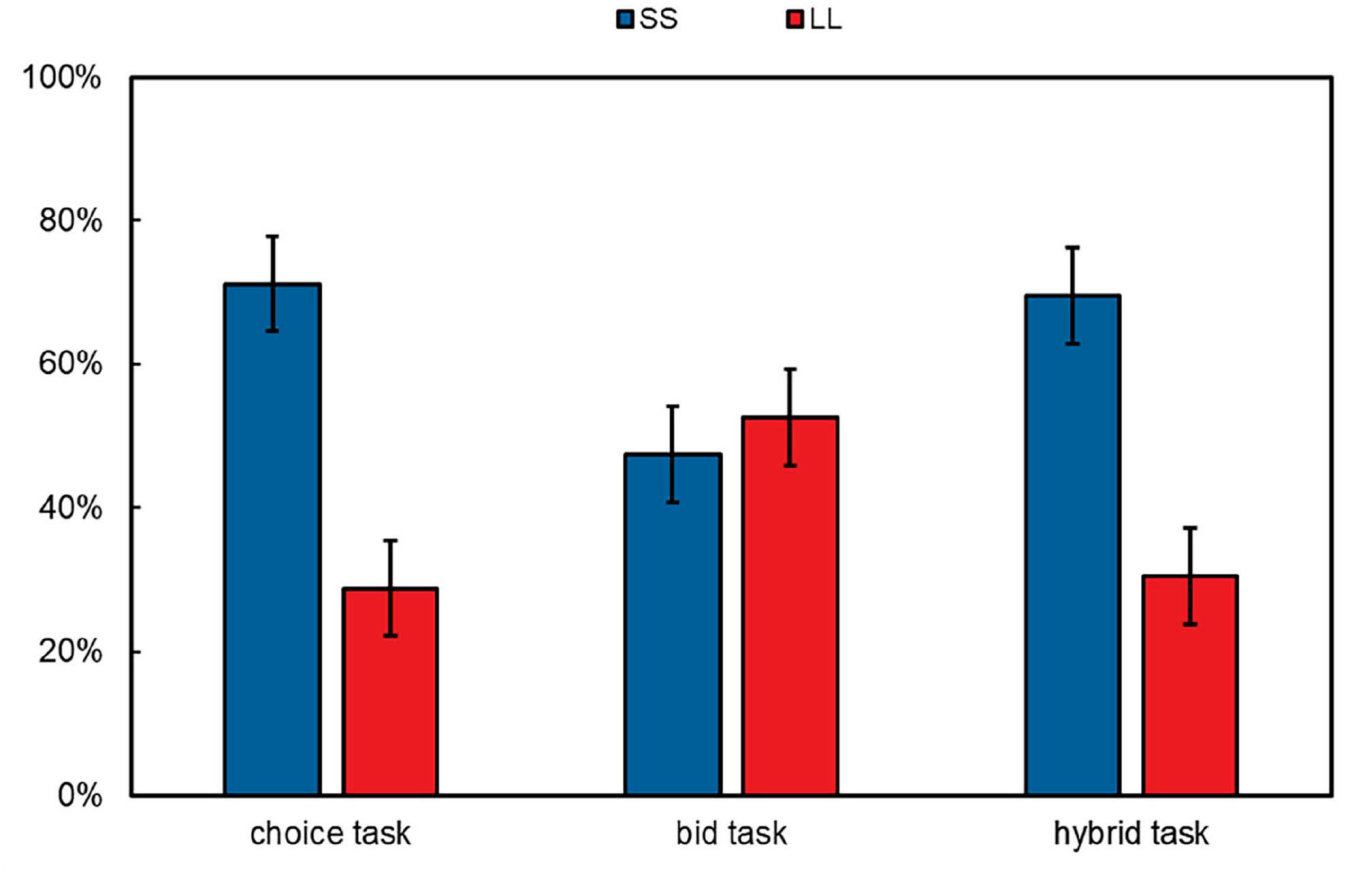
Proportions of choosing the SS options/bidding higher for the SS options (SS) and choosing the LL options/bidding higher for the LL options (LL) in the choice, bid, and hybrid tasks. Error bars represent 95% CI.

We found that the proportions of preferring LL options correlated each other in the three tasks (see Table 2). The results revealed that the proportions of choosing LL options in the choice task (*M* = 28.8%, 95% CI = [22.1%, 35.4%]) were significantly lower than those in the bid task (*M* = 52.6%, 95% CI = [45.9%, 59.2%], *t*_(52)_ = 6.08, *p* < 0.001, Cohen's *d* = 0.84, *BF* = 9.60 × 10^15^), indicating a time preference reversal effect, which is consistent with previous evidence (Tversky et al., 1990; Zhou et al., 2021). The proportions of choosing LL options in the hybrid task (*M* = 30.4%, 95% CI = [23.7%, 37.1%] did not significantly differ from those in the choice task [*t*_(52)_ = 0.72, *p* = 0.478, Cohen's *d* = 0.10, *BF* = 0.191] but were significantly lower than those in the bid task [*t*_(52)_ = −5.52, *p* < 0.001, Cohen's *d* = −0.76, *BF* = 4.53 × 10^13^], see Figure 2. The results indicated that the time preference in the hybrid task was similar to that in the choice task but different from that in the bid task.

**Table 2:** The correlation coefficient matrix of the proportions of preferring LL options in the three tasks.

**Variable**	**Choice task**	**Bid task**	**Hybrid task**
Choice task	1		
Bid task	0.32^*^	1	
Hybrid task	0.34*	0.75***	1

* *p < 0.05*,

*** *p < 0.001*.

#### 3.1.2. Response Time

**Figure 3:**
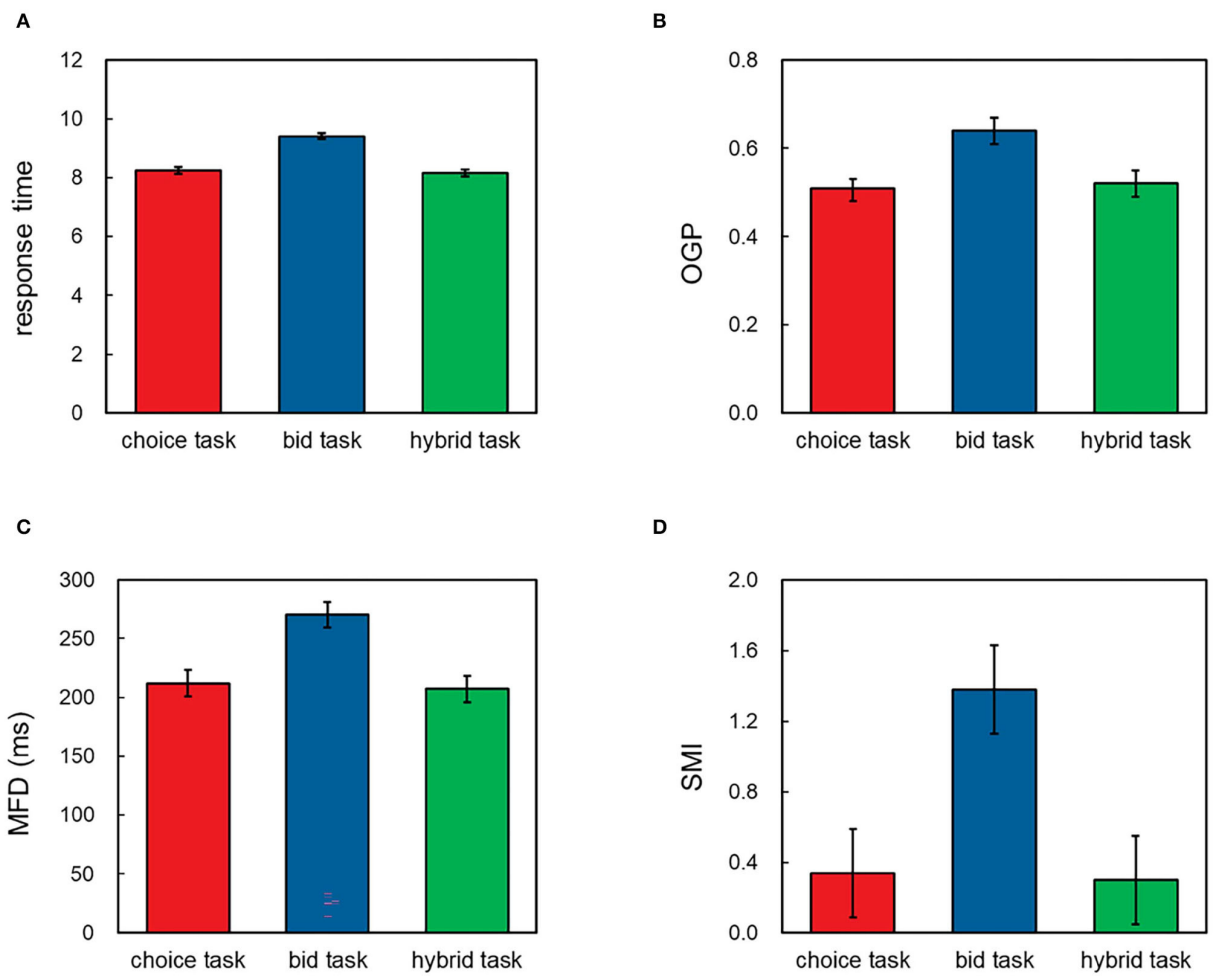
Results of **(A)** response time, **(B)** OGP, **(C)** MFD, and **(D)** SMI in the experiment. Error bars represent 95% CI.

Response times (the total amount of time a participant spent before making a choice or bid) were submitted to a natural log transformation to reduce skew. A one-way repeated-measures ANOVA conducted on the response times revealed a significant main effect of task, *F*_(2,104)_ = 249.21, *p* < 0.001, $\eta_{p}^{2}$ = 0.83, *BF* = 1.10 × 10^42^. The *post-hoc* analysis showed that the participants spent more time in the bid task (*M* = 9.41, 95% CI = [9.31, 9.52]) than in the choice task [*M* = 8.25, 95% CI = [8.14, 8.36], *t*_(52)_ = 18.07, *p* < 0.001, Cohen's *d* = 2.48, *BF* = 8.38 × 10^20^] or in the hybrid task [*M* = 8.16, 95% CI = [8.05, 8.26], *t*_(52)_ = 17.21, *p* < 0.001, Cohen's *d* = 2.36, *BF* = 9.88 × 10^19^]. No significant difference was found between the latter two tasks [*t*_(52)_ = 1.90, *p* = 0.063, Cohen's *d* = 0.26, *BF* = 0.79], see Figure 3A.

### 3.2. Eye-Tracking Results

#### 3.2.1. OGP

A one-way repeated-measures ANOVA conducted on the OGP revealed a significant main effect of task, *F*_(2,104)_ = 74.02, *p* < 0.001, $\eta_{p}^{2}$ = 0.59, *BF* = 3.32 × 10^17^. The *post-hoc* analysis showed that the participants gazed outcome attribute longer time in the bid task (*M* = 0.64, 95% CI = [0.61, 0.67]) than in the choice task [*M* = 0.51, 95% CI = [0.49, 0.54], *t*_(52)_ = 9.45, *p* < 0.001, Cohen's *d* = 1.30, *BF* = 1.15 × 10^10^] or in the hybrid task [*M* = 0.52, 95% CI = [0.49, 0.55], *t*_(52)_ = 10.11, *p* < 0.001, Cohen's *d* = 1.39, *BF* = 1.06 × 10^11^], with no significant difference between the latter two tasks [*t*_(52)_ = 0.35, *p* = 0.731, Cohen's *d* = 0.05, *BF* = 0.16], see Figure 3B.

#### 3.2.2. MFD

A one-way repeated-measures ANOVA conducted on the MFD revealed a significant main effect of task, *F*_(2,104)_ = 88.14, *p* < 0.001, $\eta_{p}^{2}$ = 0.63, *BF* = 1.32 × 10^20^. The *post-hoc* analysis showed that the values of MFD in the bid task (*M* = 270 ms, 95% CI = [259, 281] ms) were greater than those in the choice task [*M* = 212 ms, 95% CI = [201, 223] ms, *t*_(52)_ = 9.73, *p* < 0.001, Cohen's *d* = 1.34, *BF* = 2.98 × 10^10^] or in the hybrid task [*M* = 207 ms, 95% CI = [196, 218] ms, *t*_(52)_ = 10.29, *p* < 0.001, Cohen's *d* = 1.41, *BF* = 1.95 × 10^11^], with no significant difference between the latter two tasks [*t*_(52)_ = 1.65, *p* = 0.104, Cohen's *d* = 0.23, *BF* = 0.53], see Figure 3C.

#### 3.2.3. SMI

A one-way repeated-measures ANOVA conducted on the SMI revealed a significant main effect of task, *F*_(2,104)_ = 60.63, *p* < 0.001, $\eta_{p}^{2}$ = 0.54, *BF* = 1.30 × 10^15^. The *post-hoc* analysis showed that the values of SMI in the bid task (*M* = 1.38, 95% CI = [1.13, 1.63]) were greater than those in the choice task [*M* = 0.34, 95% CI = [0.09, 0.59], *t*_(52)_ = 8.35, *p* < 0.001, Cohen's *d* = 1.15, *BF* = 2.70 × 10^8^] or in the hybrid task [*M* = 0.30, 95% CI = [0.05, 0.55], *t*_(52)_ = 8.35, *p* < 0.001, Cohen's *d* = 1.15, *BF* = 2.68 × 10^8^], with no significant difference between the latter two tasks [*t*_(52)_ = 0.57, *p* = 0.570, Cohen's *d* = 0.08, *BF* = 0.18], see Figure 3D.

## 4. Discussion

The present study used eye-tracking technology to examine time preference reversal mechanisms further. By conducting a new task combining the monetary evaluation scale in the bid task and the dichotomous output format in the choice task, we tested whether the evaluation scale or the output format is the core reason for time preference reversal. Behavioral results show that participants' time preferences and response times in the hybrid task were similar to those in the choice task but different from those in the bid task. Eye-tracking measures also revealed similar patterns, indicating that the attention allocation, cognitive effort level, and information search direction in the choice and the hybrid tasks were similar but different from those in the bid task.

Our findings that a disparity in the choice and the bid tasks replicated the time preference reversal and proved its robustness. Participants chose the SS option in the choice task most of the time (71%) but assigned a higher value to the LL option in the bid task most of the time (53%) when facing the same pair of intertemporal options. This result is consistent with previous research (Tversky et al., 1990; Bohm, 1994; Zhou et al., 2021), suggesting that time preference reversal is a robust and efficient effect. Future studies may further explore the boundary of this effect.

Our findings indicated that the output format is the key reason for the time preference reversal, supporting the strategy compatibility hypothesis. In the present study, the output formats were the same in the choice and the hybrid tasks, but the evaluation scales were different between the two tasks. The similar pattern between the choice and the hybrid tasks in behavioral and eye-tracking measures indicated that the evaluation scales did not influence participants' time preference and information process. By contrast, the evaluation scales were the same in the bid and the hybrid tasks, but their output formats differed. Therefore, the discrepancy between the choice and the hybrid task in behavioral and eye-tracking measures may be because of the difference in the output formats. These findings suggested that the output format rather than the evaluation scale is the key reason for time preference reversal. Therefore, the assumption that time preference reversal is caused by different response scales, which is proposed by the compatibility hypothesis (Tversky et al., 1988, 1990; Loomes, 1990; Rubaltelli et al., 2012; Alos-Ferrer et al., 2021), is not supported by the current evidence. Instead, the present research supports the assumption that time preference reversal is caused by different (quantitative/qualitative) output formats, which is proposed by strategy compatibility hypothesis (Slovic, 1995; Stalmeier et al., 1997). To our knowledge, this study is the first to distinguish the two different theoretical hypotheses and examine the core reason for preference reversal through refined experimental design.

The eye-tracking results showed the difference in the OGP and the MFD between the choice and the bid tasks, which is helpful to understand the underlying mechanism of time preference reversals. The values of OGP can reflect the attention allocation, which is associated with decision weight (Kim et al., 2012). And the values of MFD can reflect the cognitive effort (Horstmann et al., 2009; Amblee et al., 2017). From the perspective of theoretical prediction, the scale compatibility hypothesis predicts the disparity between decision weight in the choice and the bid tasks (Tversky et al., 1988; Mellers et al., 1992), whereas the strategy compatibility hypothesis predicts the differences between the two tasks in both decision weight and cognitive effort (Stalmeier et al., 1997; Evans, 2003). Therefore, our findings are more in line with the strategy compatibility hypothesis.

This study also revealed the difference in the values of SMI between the choice and the bid tasks, indicating the difference in information search direction between the two tasks. Previous research investigating the preference reversal using an eye-tracking technique commonly constructed the bid task wherein only one option could be evaluated at a time (Kim et al., 2012; Rubaltelli et al., 2012; Alos-Ferrer et al., 2021); thus, examining the information search direction of the participants in the bid tasks becomes impossible. In the present study, the experimental stimulus in the choice and the bid tasks were visually identical, allowing us to compare the information search direction among these tasks. We found that the values of SMI in the bid task were significantly greater than those in the choice task, suggesting that more alternative-based information searches were shown in the bid task rather than the choice task. This result is consistent with the strategy compatibility hypothesis given that the deliberate strategy usually posits an alternative-based information search pattern, whereas the heuristic strategy posits an attribute-based information search pattern (Liu et al., 2021a,b). Our findings suggest that the elicitations in preference reversal may also influence the information search direction and may deepen our understanding of the mechanisms that underlie time preference reversal.

Our work also has limitations. First, the intertemporal options in the experiment only involved monetary options in a gain frame. Previous studies have shown an asymmetry between gain and loss frames in intertemporal choices (Thaler, 1981; Bilgin and LeBoeuf, 2010; Sun et al., 2015). Future studies can further examine the time preference reversals in the loss frame. Second, the statistical analysis in the present study was conducted at the individual-level and ignored the trial-by-trial variability. Future studies can use the mixed-effect regression model (Baayen et al., 2008; Judd et al., 2012; Bates et al., 2015) to control the effect of some random variables and explain the trial-by-trial variability, thus generalizing the results beyond the specific participants and items.

In summary, constructing the hybrid task enabled us to examine the hypotheses in time preference reversal and conclude that the output format is the key reason for time preference reversal, supporting the strategy compatibility hypothesis. Our findings suggest that the core reason for preference reversals might be the difference of output format between the choice and the bid tasks.

## Data Availability Statement

The datasets presented in this study can be found in online repositories. The names of the repository/repositories and accession number(s) can be found in the article/supplementary material.

## Ethics Statement

The studies involving human participants were reviewed and approved by Institutional Review Board of Nankai University. The patients/participants provided their written informed consent to participate in this study.

## Author Contributions

Y-BZ and H-ZL conceived and designed this study and wrote the article. Y-BZ, QL, and H-ZL designed experimental stimuli and procedures. Q-YL implemented experimental protocols and collected data. QL and Q-YL analyzed data. All authors contributed to the article and approved the submitted version.

## Funding

This work was partially supported by the National Natural Science Foundation of China (No. 71901126), the Humanity and Social Science Youth Foundation of Ministry of Education of China (No. 19YJC190013), and the Fundamental Research Funds for the Central Universities (No. 63212065).

## Conflict of Interest

The authors declare that the research was conducted in the absence of any commercial or financial relationships that could be construed as a potential conflict of interest.

## Publisher's Note

All claims expressed in this article are solely those of the authors and do not necessarily represent those of their affiliated organizations, or those of the publisher, the editors and the reviewers. Any product that may be evaluated in this article, or claim that may be made by its manufacturer, is not guaranteed or endorsed by the publisher.
